# Phacovitrectomy vs. consecutive vitrectomy for idiopathic macular holes: short and long-term outcomes and OCT image quality assessment

**DOI:** 10.1186/s40942-024-00614-9

**Published:** 2024-12-06

**Authors:** Vishma Prabhu, Priyanka Gandhi, Rupal Kathare, Kanika Godani, Prathiba Hande, Naresh Kumar Yadav, Jay Chhablani, Ramesh Venkatesh

**Affiliations:** 1 Dept. of Retina and Vitreous, Narayana Nethralaya, #121/C, 1st R Block, Chord Road, Rajaji Nagar, Bengaluru, Karnataka 560010 India; 2 Medical Retina and Vitreoretinal Surgery, University of Pittsburgh School of Medicine, 203 Lothrop Street, Suite 800, Pittsburg, PA 15213 United States of America

**Keywords:** Macular hole, Cataract, Outcomes, Phacovitrectomy consecutive surgery

## Abstract

**Purpose:**

To compare short- and long-term anatomical, functional, and refractive outcomes between combined phacovitrectomy (PVS) and consecutive vitrectomy (CVS) for idiopathic macular holes (MHs). Also, to evaluate the role of preoperative optical coherence tomography (OCT) image quality in guiding surgical selection.

**Methods:**

This retrospective study analyzed 183 phakic MH eyes operated between 2012 and 23, with patients divided into PVS and CVS groups. Demographic and ocular data, MH features, visual acuity (VA), refraction changes and postoperative outcomes, and follow-up details were collected. Pre- and post-operative OCT scans were evaluated for MH characteristics, OCT image quality, and surgical outcomes at short-term (≤ 3 months) and long-term (≥ 5 years) intervals.

**Results:**

The study included 144 eyes in PVS group and 39 in CVS group. Median follow-up duration was 16 months for PVS group and 72 months for CVS group (*p* < 0.001). Both groups showed significant VA improvements and comparable MH closure rates at short-term follow-up. However, CVS group had significantly better postoperative VA at short-term (*p* = 0.001) and long-term (*p* = 0.017) intervals. The preoperative OCT quality index did not significantly differ between groups and was ineffective in assessing cataract grade or guiding surgical decisions. Both groups experienced a myopic refractive shift, with a higher magnitude in the PVS group (*p* = 0.04). Postoperative complications were similar between the groups.

**Conclusion:**

CVS achieves better long-term VA than PVS following MH repair, despite similar anatomical outcomes. Preoperative OCT quality index is not effective for guiding surgical decisions, and careful refractive planning is essential, especially for PVS patients, to address postoperative myopic shifts.

**Clinical trial registration number:**

Not applicable.

## Introduction

An idiopathic macular hole (MH) is a full-thickness foveal defect that is most commonly seen in elderly females between the fifth and seventh decades of life [1–3]. At the same age, senile cataract and posterior vitreous detachment is common, making an idiopathic MH and cataract a more common co-existing pathology. Idiopathic MHs are treated surgically with pars plana vitrectomy (PPV) and release of tractional forces through posterior cortical vitreous separation and internal limiting membrane peeling, followed by gas endotamponade and face-down position [3]. In a phakic patient with a MH, PPV causes early and rapid cataract progression [4, 5]. Gas endotamponade also promotes cataract development because the gas bubble comes into contact with the posterior lens capsule, which eventually can compromise the final visual outcome [6, 7]. 

Several publications in the literature have already discussed the benefits and effectiveness of combined phacovitrectomy surgery (PVS) for MH repair by allowing clear intra-operative fundus visualization, reducing the need for future cataract surgery and thus lowering healthcare costs, and achieving early and improved post-operative visual outcomes [8–11]. Furthermore, many original research studies, as well as recent meta-analyses and systematic reviews comparing the surgical outcomes of combined PVS and vitrectomy for MH repair alone, found no significant differences in MH closure rates, visual acuity outcomes, or refractive error changes between the two groups [12–18]. As a result, many clinicians are persuaded to perform combined PVS in one session for a phakic idiopathic MH. Despite the numerous benefits of combined PVS, there is frequently post-operative posterior capsular opacification, intraocular inflammation, corneal edema, glaucoma, hypotony, and cystoid macular oedema, all of which can have a significant impact on the final visual outcomes and MH closure rates [19–22]. As a result, only eyes with a visually significant cataract and a co-existing MH should ideally be considered suitable for combined PVS.

Optical coherence tomography (OCT) is a standard imaging modality for evaluating macular pathologies, including MH, providing detailed information about MH characteristics and dimensions. Modern OCT devices offer an objective assessment of image quality through the signal-to-noise ratio (SNR), which can be adversely affected by media opacities in the cornea, lens, and vitreous [23]. This SNR ratio metric could theoretically be employed to grade cataract severity in the absence of other media opacities instead of the subjective Lens Opacities Classification System (LOCS) III grading done on slit-lamp evaluation [24, 25]. Several studies have demonstrated a significant correlation between OCT scan quality indices and cataract severity, as well as a notable influence on retinal thickness measurements [26, 27]. Consequently, we believe that OCT image quality in patients with concurrent MH and cataract may be a valuable indicator for surgeons in deciding whether to perform a combined phacoemulsification with vitrectomy or vitrectomy alone.

Several studies have reported a myopic shift in refraction following vitrectomy or phacovitrectomy, compared to cataract surgery performed for various indications [28–31]. In phakic eyes with idiopathic MHs, Liu et al. observed a significant myopic shift in postoperative refractive error compared to a control group that underwent cataract surgery for age-related cataract [32]. Multiple factors influence postoperative refraction, including axial length measurement, changes in effective lens position and anterior chamber depth, use of intraocular gas tamponade, intraocular lens power calculation formulas, and the type of the intraocular lens implanted. Additionally, phakic eyes undergoing PPV are at an increased risk of developing cataracts over time [33, 34]. Thus, the surgical sequence whether to perform combined phacovitrectomy, vitrectomy followed by subsequent cataract surgery, or cataract surgery followed by subsequent vitrectomy requires careful consideration. While most studies report a myopic shift regardless of the surgical sequence, we believe that the magnitude of this shift may differ between patients undergoing combined versus sequential procedures.

In a patient undergoing MH repair surgery, the visual acuity after surgery has been observed to stabilize over a long period of two years [35]. Demir et al. studied the long-term outcomes of combined PVS and staged MH surgeries over a 24-month period and found that both combined PVS and sequential vitrectomy, and cataract surgery were all equally safe and effective methods for treating MH and cataract [36]. However, the study findings had limitations due to the small sample size, the retrospective design, and the lack of pre-operative cataract grading. Additional research is needed to provide more information regarding the long-term anatomical and functional outcomes of patients who undergo combined PVS and sequential staged procedures for MHs.

With this study, our objective was to compare the anatomical and functional outcomes between combined and sequential vitrectomy surgery for idiopathic MHs. We also aimed to assess the associated complications, refractive changes, and long-term outcomes of each technique. Additionally, we evaluated the effectiveness of the preoperative OCT image quality index in guiding the choice of surgical procedure.

## Methods

**Fig. 1:**
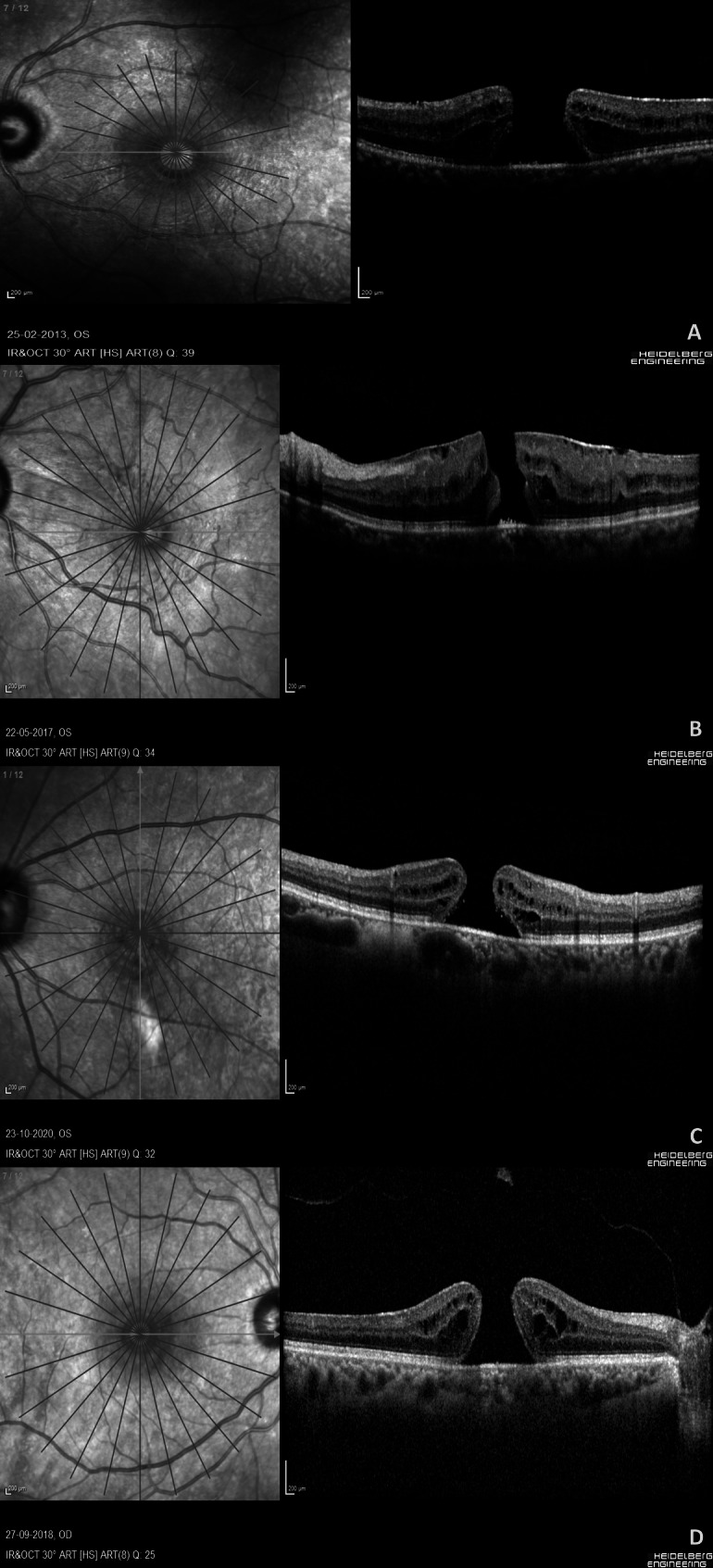
Pre operative OCT image quality scans of phakic patients with different grades of cataract undergoing macular hole repair surgery: Figure 1**A**: Pre-operative OCT image quality index of 39 in a patient with clear lens and full-thickness idiopathic macular hole (MH). Figure 1**B**: Pre-operative OCT image quality index of 34 in a patient with grade 1 nuclear sclerosis and MH with overlying epiretinal membrane. Figure 1**C**: Pre-operative OCT image quality index of 32 in a patient with grade 2 nuclear sclerosis and MH. Figure 1**D**: Pre-operative OCT image quality index of 25 in a patient with grade 3 nuclear sclerosis with posterior subcapsular cataract and MH

This single-center, retrospective, non-randomized case series analyzed phakic eyes with idiopathic MHs requiring surgical repair from 2012 to 2023. The study included eyes with stage 2, 3, or 4 MHs confirmed and staged by OCT [37, 38], excluding secondary MHs, unperformed surgeries, and cases with inadequate follow-up or post-operative OCT data. Demographic and ocular data, including age, gender, lens grading, MH characteristics, same surgeon or two different surgeons performing cataract and MH surgery during phacovitrectomy, and visual acuity outcomes, were collected. Lens grading was conducted by experienced cataract surgeons using the LOCS III during slit-lamp examination. OCT scans were acquired using the Spectralis system (Heidelberg Engineering, Germany) with a radial line scan protocol through the fovea, documenting parameters such as prefoveal vitreous attachment, epiretinal membrane presence, intraretinal cystic spaces, retinal pigment epithelium reflectivity at the fovea, and MH dimensions including outer basal diameter, minimum inner diameter, height, and left/right slopes. These measurements were used to calculate MH indices such as the hole forming factor, MH index, diameter hole index, tractional hole index, and MH area using established formulas [39]. The OCT scan quality index, a numerical value available in the scan information sheet, was recorded for the scan utilized to document MH characteristics and dimensions (Fig. 1).

Patients were divided into two groups: combined PVS or consecutive vitrectomy surgery (CVS), where cataract surgery followed MH repair. Phacoemulsification and intraocular lens implantation were performed for cataract surgery. The decision for combined surgery and the steps of MH repair were at the discretion of the treating clinician. MH surgery involved PPV with complete posterior cortical vitreous separation, internal limiting membrane peeling (peeling technique - clinician’s choice), epiretinal membrane peeling, and endotamponade with long-acting gas or silicone oil (choice of tamponade and duration of face-down positioning - clinician’s choice).

OCT scans were evaluated 1–3 months post-surgery to determine MH closure status, categorized according to Kang’s criteria [40]: (1) Closed MH, with no foveal neurosensory retinal defect; and (2) Open MH, with a residual foveal neurosensory retinal defect. Visual acuity at the last follow-up was recorded, along with the time interval between MH surgery and cataract surgery in the CVS group, and the changes in visual acuity pre- and post-cataract surgery.

Key outcomes included visual acuity, MH closure rates and refractive error changes at short-term (1-≤3months) and long-term (≥ 5years) intervals following MH surgery. The study complied with the Declaration of Helsinki, received Institutional Review Board approval, and obtained patient consent for publication while ensuring anonymity.

## Statistical analysis

Statistical analysis was conducted using GraphPad Prism v10.2.3.403 (California, USA). Data distribution was assessed with the Shapiro-Wilk normality test, and non-parametric tests were applied accordingly. Visual acuity measurements were converted from Snellen to logarithm of the minimum angle of resolution (logMAR) for analysis. Categorical variables were presented as frequencies and percentages, while quantitative variables were reported as medians with interquartile ranges or means with standard deviations. The Mann-Whitney U test and Wilcoxon signed-rank test were used for comparisons of unpaired and paired quantitative data, respectively. Fisher’s exact test was employed for categorical data comparisons between unpaired groups. The receiver operating characteristic (ROC) curve analysis was performed and the area under the ROC curve (AUC) was calculated for the pre-operative OCT image quality index to evaluate its utility in predicting the cataract grade and guiding surgical decisions. A *p*-value of < 0.05 was considered statistically significant.

## Results

### Baseline demographic and clinical characteristics

This study analyzed 183 eyes from 183 patients undergoing surgery for phakic idiopathic MHs. Of these, 144 eyes (79%) underwent combined PVS, while 39 eyes (21%) had CVS. Table 1 presents a comparison of demographic, pre-operative ocular, and OCT characteristics, as well as post-operative outcomes between the groups. Baseline demographics were comparable across groups. However, lens grading differed significantly between the two groups (*p* < 0.001). Visually significant cataracts were more commonly addressed with PVS, whereas eyes with no or early cataracts were more often treated sequentially. Eyes with nuclear sclerosis ≤ grade 2 were planned for sequential surgery. The PVS group had significantly better pre-operative visual acuity (Snellen equivalent = 20/147) compared to the CVS group (Snellen equivalent = 20/195). OCT features and MH indices were similar between the two groups at baseline.

**Table 1:** Comparison of demographic, ocular, and OCT features in combined vs. sequential phacovitrectomy for Macular holes:

Variable		PVS group (*n* = 144)	CVS group (*n* = 39)	*P* value
Age (years) [Mean ± SD]		68.35 ± 5.77	66.51 ± 5.501	0.059
Gender (M: F)		47:97	15:24	0.568
Study eye (RE: LE)		72:72	17:22	0.588
Lens grade	Clear lens (n, %)	0 (0)	12 (31)	< 0.001
	≤ NS 2 (n, %)	118 (82)	26 (67)	
	> NS2 (n, %)	12 (8)	0 (0)	
	PSC (n, %)	30 (21)	2 (5)	
	CC (n, %)	43 (30)	1 (3)	
Pre-operative LogMAR VA		0.868 ± 0.318	0.99 ± 0.339	0.043
Pre-operative refractive error (SE)		0.119 ± 1.350	-0.232 ± 1.427	0.016
Median OCT scan quality index (IQR)		26 (22–29)	27 (24–29)	0.161
OCT characteristics	Prefoveal PVD (n, %)	113 (78)	32 (82)	0.824
	ERM (n, %)	39 (27)	13 (33)	0.432
	IRC (n, %)	143 (99)	38 (97)	0.382
	Foveal RPE hyperreflectivity (n, %)	132 (92)	37 (95)	0.737
	Outer basal diameter (microns)	1090 ± 308.2	1069 ± 298.4	0.67
	Macular hole area (mm^2^)	0.31 ± 0.111	0.292 ± 0.104	0.218
	HFF	0.718 ± 0.147	0.678 ± 0.148	0.226
	MHI	0.435 ± 0.143	0.432 ± 0.143	0.951
	THI	0.242 ± 0.074	0.226 ± 0.067	0.142
	DHI	0.474 ± 0.129	0.510 ± 0.143	0.275
Median total follow-up duration (months) [IQR]		16 (3–48)	72 (30–84)	< 0.001
MH status	Closed (n, %)	123 (85)	32 (82)	0.619
	Open (n, %)	21 (15)	7 (18)	
Post-operative LogMAR VA at the last follow-up visit		0.596 ± 0.355	0.429 ± 0.419	0.001
Post-operative refractive error (SE)		-0.437 ± 0.906	-0.183 ± 1.228	0.191
Change in the refractive before and after surgery (SE)		-0.555 ± 1.490	0.05 ± 1.729	0.04

Abbreviations: M – male; F – female; PVS – phacovitrectomy surgery; CVS – consecutive vitrectomy surgery; RE – right eye; LE – left eye; NS – nuclear sclerosis; PSC – posterior subcapsular cataract; CC – cortical cataract; logMAR – logarithm of minimum angle of resolution; VA – visual acuity; SE – spherical equivalent; OCT – optical coherence tomography; IQR – interquartile range; PVD – posterior vitreous detachment; ERM – epiretinal membrane; IRC – intraretinal cysts; RPE – retinal pigment epithelium; HFF – hole forming factor; MHI – macular hole index; THI – tractional hole index; DHI – diameter hole index

### Pre-operative OCT image quality index

**Fig. 2:**
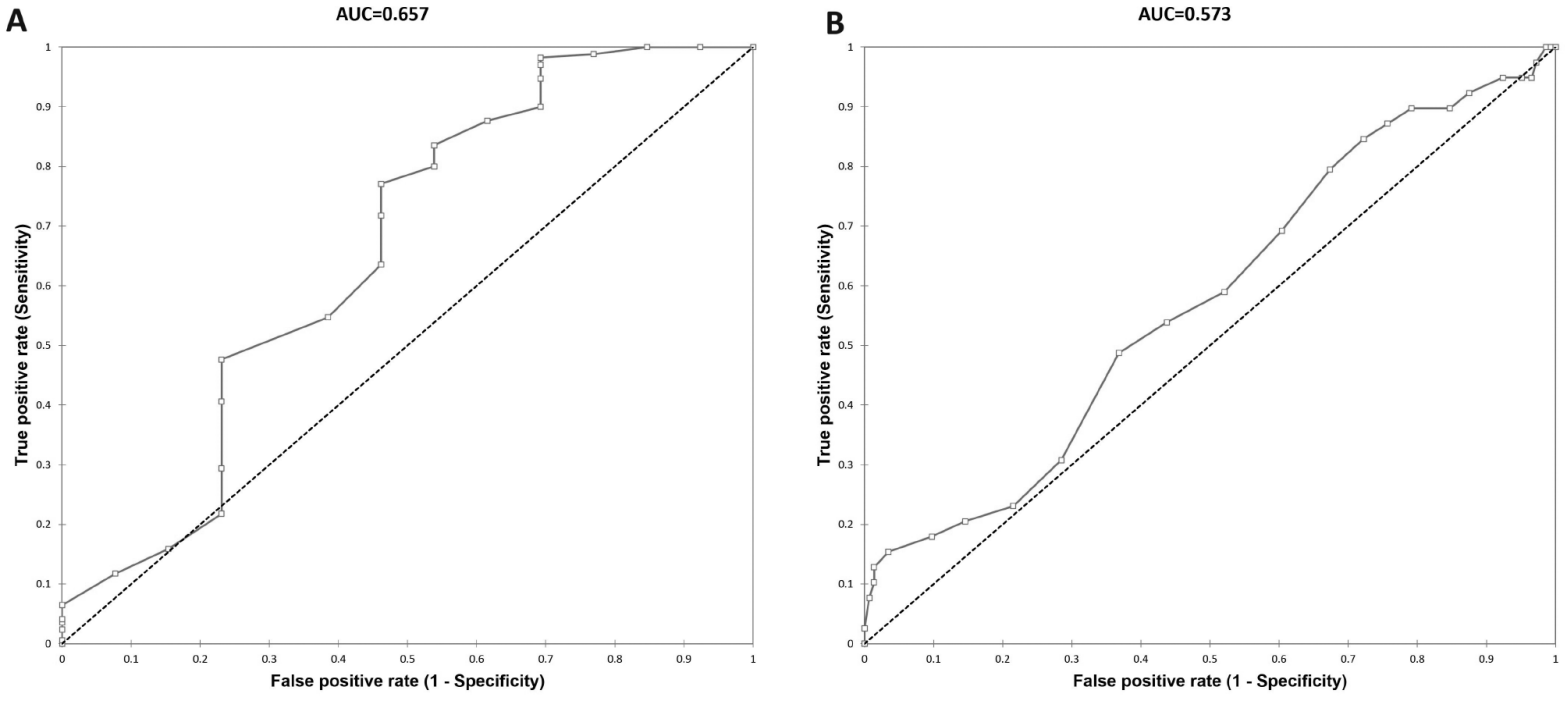
Area under ROC curve (AUC) for preoperative OCT image quality index for predicting cataract grade (Fig. 2**A**) and deciding the surgical approach (Fig. 2**B**): The AUC value was 0.657 for predicting the cataract grade and 0.573 for deciding the surgical approach

The median preoperative OCT image quality index values for the PVS and CVS groups were 26 and 27, respectively, with no statistically significant difference between the groups (*p* > 0.05). When analysing the relationship between the OCT image quality index and nuclear sclerosis cataract grade, the ROC curve yielded an AUC of 0.657. For an OCT image quality index of ≥ 23, the highest combined sensitivity and specificity was 1.309, with a predictive accuracy of 75% for the choice of surgery (*p* = 0.104). Additionally, the ROC curve was generated using the OCT image quality index as the independent variable and the surgical choice as the dependent variable, resulting in an AUC of 0.573 (Fig. 2**).** The analysis indicated that an OCT image quality index of ≤ 22 provided the highest combined sensitivity and specificity of 1.124, with a predictive accuracy of 40% for the choice of surgery (*p* = 0.157).

### Short-term post-operative anatomical, functional and refractive outcomes

Both groups exhibited significant improvements in visual acuity before and after MH surgery. Although MH closure rates were similar between groups at the last follow-up, the CVS group showed a significantly greater increase in post-operative visual acuity compared to the PVS group (20/54 vs. 20/78; *p* = 0.001). In the CVS group, the mean time from MH surgery to follow-up cataract surgery was 17.08 ± 9.696 months. Pre-cataract surgery, the mean logMAR visual acuity was 0.874 ± 0.413 (Snellen equivalent = 20/150), improving to 0.43 ± 0.418 (Snellen equivalent = 20/54) post-cataract surgery, with both paired (*p* < 0.001) and unpaired (*p* < 0.001) tests indicating significant visual acuity gains. While a myopic shift in refractive error was noted in both groups, it was not statistically significant (*p* = 0.191). However, the magnitude of myopic shift was greater in the PVS group compared to the CVS group (*p* = 0.04).

### Post-operative complications

In the PVS group, 27 (19%) eyes experienced visual acuity deterioration at the final follow-up. The causes included significant posterior capsular opacification in 11 (41%) eyes, failed MH closure in 7 (26%) eyes, combined Brilliant blue G (BBG) and endoillumination toxicity in 4 (15%) eyes, retinal detachment in 3 (11%) eyes, and cystoid macular edema in 2 (7%) eyes. In the CVS group, 9 (23%) of 39 eyes had decreased visual acuity, attributed to posterior capsular opacification, failed MH surgery, elevated intraocular pressure, and retinal detachment (2 eyes each, 22%), and combined BBG and endoillumination toxicity (1 eye, 11%). Risk ratios comparing the PVS to CVS group for complications were as follows: posterior capsular opacification 1.216 (95% CI: 0.789–1.788), failed MH closure 1.05 (95% CI: 0.593–1.517), combined BBG and endoillumination toxicity 1.078 (95% CI: 0.496–1.537), retinal detachment 0.775 (95% CI: 0.294–1.253), and cystoid macular edema 1.36 (95% CI: 0.460–1.664).

### Long-term (≥ 5 years) follow up outcomes

Long-term follow-up data (≥ 5 years) were available for 33 (23%) eyes in the PVS group and 24 (62%) eyes in the CVS group. The rates of MH closure did not differ significantly between the groups during the extended follow-up period (*p* = 0.728). However, final visual acuity was significantly better in the CVS group (mean logMAR = 0.333 ± 0.385; Snellen equivalent = 20/43) compared to the PVS group (mean logMAR = 0.481 ± 0.349; Snellen equivalent = 20/61) (*p* = 0.017).

## Discussion

This study demonstrated that the combined PVS and CVS groups had comparable anatomical outcomes, refractive changes, and complication rates. However, the CVS group exhibited significantly greater improvement in visual acuity and similar MH closure rates compared to the PVS group, even with a follow-up period of at least 5 years. Additionally, the preoperative OCT image quality index was found to be ineffective in corelating with the cataract grade and guiding clinicians on the optimal timing for cataract surgery in patients undergoing MH surgery. In addition, the degree of myopic shift was greater in the PVS group.

For over a decade, numerous studies have demonstrated the advantages of simultaneous PVS over sequential surgery for managing MHs in phakic patients [8–11]. This approach is now favoured by clinicians due to its benefits of faster visual recovery, reduced need for future cataract surgery, and lower surgical costs. Consequently, our study also found a significantly higher proportion of cases were managed with combined PVS rather than sequential surgeries.

In this study, except for preoperative visual acuity and lens status, demographic, ocular, and OCT features were similar between the PVS and CVS groups. The PVS group demonstrated significantly better preoperative visual acuity compared to the CVS group. Notably, a substantial proportion of eyes with early nuclear cataracts underwent simultaneous vitrectomy, although cataract surgery could have been delayed in many instances. The literature consistently highlights the advantages of combined PVS, encouraging retinal surgeons to favour this approach over sequential surgeries, even for early cataract signs [8–11]. Consequently, the PVS group exhibited shorter follow-up intervals and higher preoperative visual acuity than the CVS group.

This study revealed a notable difference in postoperative visual acuity gains between the two groups. Both groups experienced improvement, but the CVS group achieved better mean visual acuity at the last follow-up visit than the PVS group, despite having poorer mean preoperative visual acuity. This discrepancy is attributed to a significant number of eyes with early cataracts undergoing a two-step procedure. Although MH closure was successful with similar complication rates in both groups, cataract progression in the CVS group likely led to reduced visual acuity. Consequently, the CVS group saw significantly greater visual acuity improvement post-cataract surgery. These findings contrast with existing literature, which reports similar visual gains and MH closure rates for combined PVS and CVS groups [12–14]. In a study of 120 eyes with idiopathic MHs and cataracts treated with one- or two-step procedures, the authors found no significant improvement in far visual acuity in the consecutive surgery group at 6 months, while the combined surgery group showed significant improvement. Closure rates were 100% and 96% in the combined and consecutive groups, respectively, with no significant difference in complication rates [15]. 

The presence and grade of cataract are known to influence OCT imaging, often resulting in poor preoperative image quality [27, 41]. We aimed to utilize the OCT image quality index to determine cataract grade and plan the appropriate surgical procedure. However, our findings showed no significant difference in the OCT image quality index between the PVS and CVS groups, rendering it ineffective for cataract grading and surgical planning. OCT image quality can be affected by factors other than cataract grade, such as patient’s age, pupil size, other eye opacities, and vitreous humor opacities leading to decreased effectiveness [42, 43]. 

Our study confirmed a myopic shift in refractive error in both the PVS and CVS groups, aligning with previous findings in the literature. Notably, the PVS group demonstrated a more significant refractive change compared to the CVS group. This is an important observation in the study, especially for patients planning cataract surgeries with premium-quality and high-cost intra ocular lenses. This pronounced myopic shift in the PVS group may be attributed to initial inaccuracies in refraction measurements due to the presence of MH. Post-vitrectomy and membrane peeling, the normalization of macular morphology could contribute to this myopic shift, similar to changes observed following treatment of macular edema with pharmacological agents or resolution of neurosensory fluid in central serous chorioretinopathy [44, 45]. Additional factors such as alterations in corneal curvature, variations in anterior chamber depth, and scleral stretching and thinning associated with combined PVS may further account for the greater myopic shift observed in this group compared to sequential surgery [31, 32, 34]. 

Several additional factors can influence the decision to choose a specific surgical approach when treating a phakic MH, including the availability of retinal surgeons, quality instrumentation, and insurance disbursement policies, particularly in peripheral centers and smaller eye hospitals. These factors may affect whether a single surgeon or two different specialists are involved in performing the cataract and MH surgeries during combined phacovitrectomy surgery (PVS). In this study, combined PVS procedures were performed by two different surgeons, each specializing in either cataract surgery or pars plana vitrectomy (PPV). While this dual-specialist approach allows patients to benefit from specialized expertise, it introduces logistical challenges such as scheduling and operating room coordination. The literature lacks direct comparisons of anatomical, functional, and refractive outcomes between combined procedures performed by a single surgeon versus those involving multiple surgeons. Existing studies indicate that having a single surgeon perform both procedures is safe and effective [8, 12–14, 34]. However, our cohort did not include cases performed by a single surgeon, precluding a comparative analysis based on the number of surgeons involved. From a cost-effectiveness perspective, the combined PVS group demonstrated lower overall costs compared to the sequential vitrectomy and phacoemulsification group [16]. This cost advantage could be leveraged by insurance companies to reduce procedural expenses and potentially increase approvals for combined PVS procedures.

The limitations of this study included a retrospective design, an uneven distribution of cases between the two groups, a single surgeon not performing both cataract surgery and MH surgery in the PVS group, and the presence of numerous factors that could potentially affect anatomical, functional, and refractive outcomes. Nevertheless, this study yielded several significant insights. The study found that the pre-operative OCT image quality index did not effectively guide the surgical approach. However, it was shown that using a consecutive surgery approach resulted in better visual gains and long-term outcomes in a large group of patients with phakic idiopathic MH eyes. Furthermore, the significant myopic shift in the combined PVS group may make clinicians wary of recommending premium intraocular lenses during cataract management.

In conclusion, this study showed that the sequential group achieved greater and more sustainable visual acuity improvement compared to the PVS group, despite similar anatomical and refractive outcomes at the short-term and long-term follow-up intervals. The preoperative OCT image quality index was ineffective for surgical planning. Future randomized clinical trials with balanced case distribution are needed for more definitive conclusions.

## Data Availability

No datasets were generated or analysed during the current study.

## Declarations

### Animal research

“This article does not contain any studies with animals performed by any of the authors.”

## Abbreviations

MH: Macular hole
OCT: Optical coherence tomography
BBG: Brilliant blue G
PPV: Pars plana vitrectomy
SNR: Signal–to– noise ratio
PVS: Phacovitrectomy surgery
CVS: Consecutive vitrectomy surgery
LOCS: Lens opacities classification system
LogMAR: Logarithm of minimum angle of resolution
ROC: Receiver operating characteristics
AUC: Area under curve
